# Comprehensive Nutritional and Functional Characterization of Novel Mycoprotein Derived from the Bioconversion of *Durvillaea* spp.

**DOI:** 10.3390/foods13152376

**Published:** 2024-07-27

**Authors:** Catalina Landeta-Salgado, Nicolás Salas-Wallach, Javiera Munizaga, María Paz González-Troncoso, César Burgos-Díaz, Lhaís Araújo-Caldas, Patricia Sartorelli, Irene Martínez, María Elena Lienqueo

**Affiliations:** 1 Department of Chemical Engineering, Biotechnology, and Materials, Center for Biotechnology and Bioengineering (CeBiB), University of Chile, Beauchef 851, Santiago 8370456, Chile; n.salas.wallach@gmail.com (N.S.-W.); jmmunizaga@uc.cl (J.M.); imartinez@ing.uchile.cl (I.M.); mlienque@ing.uchile.cl (M.E.L.); 2 Agriaquaculture Nutritional Genomic Center, CGNA, Temuco 4780000, Chile; cesar.burgos@cgna.cl; 3 Instituto de Ciências Ambientais, Químicas e Farmacêuticas, Universidade Federal de São Paulo, Diadema Campus, Sao Paulo 09913-030, SP, Brazil; lhaisaraujocaldas@gmail.com (L.A.-C.); psartorelli@unifesp.br (P.S.)

**Keywords:** mycoprotein, seaweed, *Durvillaea* spp., biocompounds, nutritional composition, metabolome, bioactivity

## Abstract

This study aimed, for the first time, to determine the nutritional composition, beta-glucan and ergosterol contents, phenolic compound composition, and biological and functional activities of a novel mycoprotein produced through a bioconversion process of *Durvillaea* spp., a brown seaweed. An untargeted metabolomics approach was employed to screen metabolites and annotate molecules with nutraceutical properties. Two products, each representing a distinct consortia of co-cultured fungi, named Myco 1 and Myco 2, were analysed in this study. These consortia demonstrated superior properties compared to those of *Durvillaea* spp., showing significant increases in total protein (~238%), amino acids (~219%), and β-D-glucans (~112%). The protein contains all essential amino acids, a low fatty acid content, and exhibits high antioxidant activity (21.5–25.5 µmol TE/g). Additionally, Myco 2 exhibited the highest anti-alpha-glucosidase activity (IC_50_ = 16.5 mg/mL), and Myco 1 exhibited notable anti-lipase activity (IC_50_ = 10.5 mg/mL). Among the 69 top differentially abundant metabolites screened, 8 nutraceutical compounds were present in relatively high concentrations among the identified mycoproteins. The proteins and polysaccharides in the mycoprotein may play a crucial role in the formation and stabilization of emulsions, identifying it as a potent bioemulsifier. In conclusion, the bioconversion of *Durvillaea* spp. results in a mycoprotein with high-quality protein, significant nutritional and functional value, and prebiotic and nutraceutical potential due to the production of unique bioactive compounds.

## 1. Introduction

Rapid demographic growth has spurred interest in sustainable, healthy alternative protein sources to meet global demands, necessitating a transformation of the food system to link diet with health and environmental sustainability [1,2]. Alternative proteins aim to replace traditional animal products by matching or exceeding them in flavour and cost-effectiveness, but none have a perfectly replicated taste or reduced cost, leading researchers to explore fungi as promising alternatives [1,3]. Fungi are considered a promising source of vegan protein due to their high protein content and complete amino acid profiles [4].

Recent studies have demonstrated that various strains of filamentous fungi can be cultivated in submerged liquid cultures and harvested at the mycelial stage to produce a high biomass content or mycoprotein content [5,6], a variety of prebiotic compounds (β- and α-glucans) [7], and bioactive compounds such as proteins, enzymes, lipids, phenolic derivatives, sterols, and carbohydrates [8,9]. One well-known example is Quorn, produced by Marlow Foods^®^, which utilizes the entire biomass of *Fusarium venenatum*, a fungus cultivated in continuous fermentation with high-quality carbohydrates and micronutrients to create protein-rich, alternative meat products [10]. Another example is the mycoprotein derived from *Neurospora crassa*, which is produced through fermentation and minimal downstream processing and has demonstrated both safety and nutritional value [11]. In Sweden, Mycorena^®^ produces a mycoprotein with a neutral taste and meat-like texture through a controlled fermentation process [12]. In addition, Fermotein^®^, a mycoprotein developed from the thermophilic fungus *Rhizomucor pusillus*, has a simple, cost-efficient, scalable, and energy-efficient production process [13]. Additionally, a nontoxic strain of *Penicillium limosum* with high biomass yield, protein content, and essential amino acid content has been isolated from wheat, providing another promising mycoprotein source [14]. There is abundant evidence supporting the nutritional and nutraceutical content of fungal mycelia. Consuming this food is associated with numerous health benefits, including cholesterol reduction, antidiabetic activity, and immunomodulatory effects [15,16]. Controlling cultivation conditions in bioreactors for mycelium production makes submerged fermentation (FSm) more reliable, efficient, and safe than fruiting body production in solid-state fermentation [8,17]. An alternative for mycelial biomass production is the fermentation or bioconversion of agri-food byproducts or other types of biomass, such as seaweed, to add nutritional value to the substrate [18,19,20,21]. Seaweeds are a rich resource of complex carbohydrates (alginate, cellulose, and agar, among others) that can be assimilated by a group of filamentous fungi. These fungi can depolymerize and assimilate these carbohydrates with the help of specific enzymes [21,22,23,24].

In addition, seaweeds are perceived as healthy by consumers and are rich in nutrients such as lipids, carbohydrates, vitamins, minerals, and biologically active compounds, which have nutritional relevance and health benefits [25]. Their production is more sustainable than that of terrestrial crops, especially brown algae, which are the fastest-growing linear organisms, expanding up to 60 cm per day and allowing multiple harvests per year. These algae also play a crucial role in ecosystem development and global warming mitigation [26,27]. Species of the genus *Durvillaea*, found solely in the Southern Hemisphere, can grow over 10 m in length and are important dispersal agents for various species along the cold-temperate coasts of Chile [28,29]. Indeed, *D. incurvata* and *D. antarctica*, also known as Cochayuyo, are popular edible seaweeds in Chile that are commonly used as gastronomic ingredients in salads, soups, side dishes, and entrees [30,31].

Mycoseaweed^®^, a FoodTech company from Chile specializing in the development of mycoproteins, has focused on producing mycelial biomass through submerged fermentation of a consortia of edible fungi, using *Durvillaea* spp. as the sole carbon source. This process not only develops innovative mycoprotein products but also valorises brown seaweeds. This article aimed to explore the potential of a novel mycoprotein by analysing and evaluating two products of Mycoseaweed^®^, each representing a different consortia of co-cultured fungi, named Myco 1 and Myco 2, and comparing them with the nutritional properties of *Durvillaea* spp. This investigation included nutritional characterization studies, techno-functionality analysis (emulsifying properties), and evaluations of metabolites of nutritional interest or bioactive compounds. Additionally, assessments of the antioxidant properties, along with α-glucosidase and lipase inhibitory activities, were conducted for these products. Our study is novel in demonstrating the comprehensive nutritional and functional characterization of mycoprotein derived from the bioconversion of *Durvillaea* spp. This research highlights the bioactive compounds and potential health benefits associated with this innovative mycoprotein source, providing a foundation for future developments in functional and nutraceutical foods.

## 2. Materials and Methods

### 2.1. Production of the Alternative Protein or Mycoprotein

Mycoseaweed^®^ has devised a technique to produce mycoprotein, an alternative protein from mycelium, using consortia (co-cultures) developed with different filamentous fungi and with brown seaweed as the sole carbon source. The production of two kinds of consortia named Myco 1 and Myco 2, involved the aseptic preparation of sterile growth media supplemented with *Durvillaea* spp. as the sole carbon source. This medium was then inoculated with liquid fungal cultures of the genera *Pleurotus*, *Lentinula*, *Hericium*, and *Ganoderma*, with final concentrations between 10% and 15% *v*/*v*. The culture was developed in a controlled bioreactor using submerged fermentation with constant agitation and aeration to foster a dense, highly dispersed suspension of mycelial biomass. After growth, the mycelia were harvested, rinsed, filtered, freeze-dried, and milled into fine flour. The pulverized seaweed was donated by Herbamar™, a company based in Concepción, Chile.

### 2.2. Nutritional Characterization of the Raw Material

#### 2.2.1. Proximal Composition

The moisture content was measured gravimetrically, the ash content was determined by incinerating the sample at 550 °C for 6 h, and the fat content was analysed via Soxhlet extraction. Total protein was quantified using the Kjeldahl method, with a conversion factor of 6.25 applied to convert the nitrogen content to protein. Crude fibre was assessed using a gravimetric-chemical method (AOAC, 2000). The carbohydrate content was estimated by subtracting the cumulative mass of protein, moisture, fat, fibre, and ash from the total mass [32].

#### 2.2.2. Analysis of Amino Acids

The amino acid profiles of the seaweed biomass, Myco 1, and Myco 2 samples were determined by liquid-phase acid hydrolysis. Hydrolysis and derivatization were based on the procedure previously described by Astorga-España et al. [33] using an AccQ·Fluor Reagent Kit (WAT052880, Waters Corporation, Milford, MA, USA). The sample was analysed using an HPLC system with a fluorescence detector (Shimadzu, Kyoto, Japan) and an AccQ-Tag Amino Acids C18 reversed-phase column (60 Å, 4 µm, 3.9 mm × 150 mm; Waters Corporation, USA). The quantification was carried out using an external amino acid standard H (NCI0180; Thermo Scientific™, Rockford, IL, USA). Chromatographic separation was performed following the method previously described by [34] with slight modifications. Mobile phase A consisted of 140 mM sodium acetate, 20 mM TEA, and 3.42 mM EDTA in water titrated to pH 5.02 with phosphoric acid. Mobile phase B was 60% acetonitrile in water (*v*/*v*).

#### 2.2.3. Ergosterol Content

Extraction was carried out by vortexing 0.1 g of mycelium with hexane (1:60 *w*/*v*), for 2 min followed by incubation for 2 min on ice. The extract was centrifuged at 6000× *g* at 4 °C, for 15 min. The extraction process of the resulting pellet was repeated, and the resulting supernatants were combined and filtered through a 0.22 µm PTFE filter. The extract was concentrated using a nitrogen flow at 40 °C and reconstituted in 500 µL of methanol. The sample was analysed using an HPLC system with a photodiode array (Shimadzu, Kyoto, Japan) and an Onyx monolithic C18 reversed-phase column (5 µm × 100 mm and 4.6 mm, Phenomenex, Torrance, CA, USA). Chromatographic separation was performed following the method previously described by Souilem et al. [35]. The concentration of ergosterol in the samples was estimated using an external standard curve of ergosterol (Cod. PHR1512, Sigma Aldrich, Saint Louis, MO, USA) at concentrations between 0.1 and 0.3 mg/mL

#### 2.2.4. Estimation of β-Glucan Content

The analysis of the (1,3)-(1,6)-β-glucan content was conducted using a Megazyme Yeast and Mushroom Kit (K-YBGL) (Megazyme Ltd., Bray, Co. Wicklow, Ireland) following the manufacturer’s protocol [36]. Initially, samples (*Durvillaea* spp. Myco 1 and Myco 2) were milled and then treated with 12 M H_2_SO_4_ at 4 °C for 2 h to solubilize the glucans. This was followed by hydrolysis in 2 M H_2_SO_4_ at 100 °C for another 2 h. After hydrolysis, any residual glucan fragments were completely hydrolysed to glucose by employing a mixture of exo-1,3-β-glucanase and β-glucosidase, allowing for total glucan content measurement. To determine the alpha-glucan and sucrose contents, specific hydrolysis of D-glucose and D-fructose was performed. Glucose levels were quantified using amyloglucosidase and invertase, followed by a glucose oxidase-peroxidase (GOPOD) reagent. The β-glucan content was subsequently calculated by subtracting the measurements of glucose from the total glucan quantified.

### 2.3. Total Phenolic Compounds

#### 2.3.1. Preparation of Extracts

Phenolic extracts of seaweed biomass, Myco 1, and Myco 2 were prepared using 70% ethanol (1:10 *w*/*v*) added to 0.3 g of sample. The mixture underwent five cycles of vortexing, ultrasonication in a cleaner bath (SB-5200DTD, Scientz, Ningbo, Zhejiang, China), and incubation on ice for 2, 5, and 15 min, respectively. Then, the ethanol mixtures were incubated overnight at 4 °C. The extracts were later centrifuged at 10,500× *g* at 4 °C, for 15 min, after which the supernatants were collected. The residues were extracted under the same conditions, and then the collected supernatants were combined and filtered with Whatman^®^ qualitative Grade 1 filter paper (WHA1001150, Sigma Aldrich, USA). The extracts were concentrated using a nitrogen flow at 35 °C and then freeze-dried [37].

#### 2.3.2. Total Phenolic Content

The phenolic extracts were dissolved in 70% ethanol to a concentration of 10 mg/mL. Phenolic extracts were estimated by the Folin–Ciocalteu protocol previously described by Chew et al. [38] with several modifications. Briefly, the samples and standard (100 µL) were mixed with 500 µL of 0.2 N Folin–Ciocalteu′s phenol reagent (F9252, Sigma Aldrich, USA) and incubated at room temperature for 4 min. Then, 500 µL of 0.7 N sodium carbonate solution was added to the mixture and incubated in a dark environment at room temperature for 2 h. The absorbance was measured at 760 nm using a SPECTROstar Omega spectrophotometer (BMG LABTECH, Ortenberg, Germany). The total phenolic content was expressed in milligrams of gallic acid equivalents (GAE) per 100 g of dry weight (dw).

#### 2.3.3. Identification of Phenolic Compounds

The extraction and identification were carried out following the protocol previously described by Noriega et al. [39] with several modifications. Briefly, chromatographic separation was performed with a Kromasil C-18 column (250 mm × 4.6 mm, 5 µm particle size), mobile phase A (0.1% *v*/*v* formic acid in water), and mobile phase B (acetonitrile). The gradient conditions were as follows: 0.00–2 min, 94% A; 2.01–45.00 min, 94–75% A; 45.01–56 min, 75–40% A; 57.01–59 min, 40–94% A; and 59.01–65 min, 94% A.

### 2.4. Bioactivity Assay

#### 2.4.1. In Vitro Antioxidant Activity

The antioxidant capacities of the Myco 1, Myco 2, and seaweed extracts (1 mg/mL) were determined using a Total Antioxidant Capacity Assay Kit (MAK187, Sigma Aldrich, USA) [40]. The extract samples with 100 µL of Cu^2+^ Working Solution were mixed and incubated for 90 min at room temperature. The absorbance was measured at 570 nm using a SPECTROstar Omega spectrophotometer (BMG LABTECH, Germany). The results are expressed as micromoles of Trolox equivalent antioxidant activity per gram of sample (µmol TE/g).

#### 2.4.2. α-Glucosidase Inhibitory Effect

The analysis was carried out following a variation of the protocol described by Costamagna et al. [41]. A reaction mixture of 175 μL of sodium phosphate buffer (pH 7.4), 25 μL of enzyme (1 U/mL) and 25 μL of sample (100 mg/mL) was incubated for 15 min at 37 °C. Then, the enzymatic reaction was started by adding 25 μL of 5 mM p-nitrophenyl α-d-glucopyranoside. The absorbance was measured at 405 nm every min for 20 min. IC_50_ values denote the mg GAE/mL required to inhibit the enzyme by 50%.

#### 2.4.3. Lipase Inhibitory Activity

The assay was carried out according to a previously described protocol by López-Belchí et al. [42], with modifications. Lipase solution (50 μL, 10 mg/mL) was mixed with 150 μL of sodium phosphate buffer (pH 8) and 25 μL of sample and incubated at 37 °C for 15 min. Then, 25 μL of p-nitrophenyl dodecanoate (5 mM) was added to begin the enzyme reaction. The absorbance was measured at 405 nm every min for 25 min. IC_50_ values denote the mg GAE/mL required to inhibit the enzyme by 50%.

### 2.5. Untargeted Metabolomics Using UPLC-Q-TOF-HRMS/MS

The samples were analysed by ultra-performance liquid chromatography quadrupole-time-of-flight mass spectrometry using an Elute System (Bruker Daltonics Inc., Bremen, Germany).

#### 2.5.1. Extraction

The samples (0.3 g) with 70% methanol (1:10 *w*/*v*) were sonicated using a Q500 Sonicator^®^ (QSonica, Newtown, CT, USA) for 5 min (cycles of 10 s on, 10 s off, and 40% amplitude) with a 3.2 mm diameter probe [37]. The mixture was then incubated with agitation overnight at RT and 150 rpm. The extracts were centrifuged at 4000× *g* for 10 min at 4 °C, after which the supernatants were collected. The residues were re-extracted, and the combined supernatants were filtered with Whatman^®^ 1 filter paper. The solvent was evaporated by applying a nitrogen flow at 35 °C and then freeze-dried.

#### 2.5.2. UHPLC-MS/MS Data Acquisition and Molecular Networking

The extract was then diluted with 70% methanol for a final concentration of 1 mg/mL and submitted to UPLC-HRMSMS analysis [43]. The separation was performed on a Kinetex C18 column (100 mm × 2.1 mm, 1.7 μm particle size) with a flow rate and sample injection of 0.4 mL/min and 10 μL, respectively, and a column temperature of 40 °C. Mobile phases A (0.1% *v*/*v* formic acid in water) and B (0.1% *v*/*v* formic acid, 90% *v*/*v* acetonitrile in water) were used. The initial conditions were 22% B for 1 min. The gradient conditions were elevated from 22% to 99% B in 10 min, maintained at 99% B for 2.5 min, decreased to 22% B in the next 0.5 min, and held there for 3 min. Mass spectrometry data were acquired over a range of *m*/*z* 50–1300 in the positive and negative ion mode of the electrospray ionization (ESI) source: capillary voltage 4500 V, pressure 2 bar, dry gas temperature 250 °C, and dry gas flow rate 8 L/min. MetaboScape^®^ 4.0 software (Bruker Daltonics Inc., Bremen, Germany) was used for data acquisition and processing. Global Natural Products Social Molecular Networking (GNPS; http://gnps.ucsd.edu (accessed on 17 march 2024)), an open-access knowledge base for community-wide organization and sharing of raw materials, was used for the identification of *tandem* mass spectrometry (MS/MS) data [44]. All HRMS/MS data obtained from the analysis of extracts were converted to the “.mzML” extension using the free software MSConvert (Proteowizard 3.0.21229^®^). The “.mzML” data were then uploaded to the Mass Spectrometry Interactive Virtual Environment (MassIVE) Web server using WinSCP to create the molecular networking using the GNPS platform, as well as to perform the dereplication for database matches. To create the molecular networking, the acquired data were treated in the GNPS Data Analysis platform removing fragments of ±17 Da of precursor *m*/*z*. The basic options for mass tolerance ions were set to 0.02 Da for precursor and QTOF fragment ions. A network was then created using the MS-Cluster algorithm according to a cosine score above 0.7, more than 2 matched peaks, and minimum cluster size for 1 spectrum. The spectra in the network were then searched against GNPS’ spectral libraries. The library spectra were filtered in the same manner as the input data. All matches kept between network spectra and library spectra were required to have a score above 0.6 and at least 3 matched peaks. The software Cytoscape 3.10.2^®^ was used to visualize and edit the entire molecular networking, as well as on the GNPS website.

### 2.6. Techno-Functional Properties

#### 2.6.1. Emulsifying Capacity (EC) and Emulsion Stability (ES)

The emulsifying capacity (EC) and emulsion stability (ES) were determined according to Opazo-Navarrete et al. [45] with minor modifications. Thus, 0.2 g of sample was added to 20 mL of distilled water (1:100 *w*/*v*) in a 50 mL Falcon tube and shaken at room temperature for 1 h through constant stirring using a magnetic stirrer. Furthermore, the sample was adjusted to pH 7, 20 mL of sunflower oil was added (1:1 *v*/*v*), and the sample was homogenized for 3 min at 10,000 rpm utilizing a high speed homogenization, Ultra-Turrax (IKA-Werke GmbH & Co. KG, Staufen, Germany). Then, the sample was allowed to stand at room temperature for 1 h. The emulsions were transferred to test tubes, and the total and emulsion layer heights were measured at 0 and 24 h. The EC_24_ was estimated as the relation between the height of the emulsion layer at 24 h (H_EL_) and the total height of the sample (H_T_). Additionally, the ES was calculated by dividing the EC_24_ by the EC at the start time (EC_0_).

$$\mathrm{EC}\left(\%\right)=\frac{H_{\mathrm{EL}}}{H_{T}}\times 100$$

$$\mathrm{ES}\left(\%\right)=\frac{{\mathrm{EC}}_{24}}{{\mathrm{EC}}_{0}}\times 100$$

#### 2.6.2. Microstructure Observation of the Emulsions

The microstructure of the emulsions stabilized with the different concentrations of Mycoseaweed’s mycoprotein was visualized using an optical microscope (Olympus-BX40, Tokyo, Japan) equipped with a camera to estimate the droplet size and aggregate state.

### 2.7. Statistical Analysis

Statistical analysis was performed using GraphPad Prism 10 (GraphPad Software, San Diego, CA, USA). The experiments were conducted in triplicate, and the results are expressed as the mean ± standard deviation. One-way analysis of variance (ANOVA) was performed using Statgraphics Centurion v.19 statistical software (Statpoint Technologies Inc., Warrenton, VA, USA). Differences were considered significant at *p* < 0.05 according to Tukey’s test.

## 3. Results and Discussion

### 3.1. Nutritional Components

The chemical compositions of Myco 1 and Myco 2 indicate that these mycoproteins are high in protein and low in fat. To provide a clear understanding of the workflow and key outcomes, a detailed schematic diagram is included (Figure S1; Supplementary Materials). The nutritional profiles of Myco 1, and Myco 2 are detailed in Table 1. Notably, the protein content significantly increased from 10.79 ± 0.08 g/100 g in *Durvillaea* spp. [46], to 36.5 ± 0.189 g/100 g and 33.8 ± 0.170 g/100 g in Myco 1 and 2, respectively, representing an ~3.3-fold increase. Significant differences were also observed in the protein content between Myco 1 and Myco 2 (Table 1).

These fungi secrete extracellular enzymes that hydrolyse complex polymers in brown algae, releasing carbon, nitrogen, and other essential nutrients for growth and metabolism. Previous studies have demonstrated the ability of fungi to increase protein levels in agro-industrial wastes and algae [20,21,47,48]. The increase in protein content is likely due to enzymatic degradation of polymers into mono- or short-chain oligomers, facilitating nutrient assimilation, including nitrogenous compounds, and promoting changes in protein solubility and the nitrogen concentration [49,50]. Additionally, this process degrades carbohydrates, converting them into fungal biomass and CO_2_ [51].

In terms of the carbohydrate content, significant differences were observed compared to those of *Durvillaea* spp. (~54.57 g/100 g) (Table 1) [46]. Both Myco 1 and Myco 2 showed a decrease in the carbohydrate content (41.19%) relative to *Durvillaea* spp. [46]. Additionally, approximately 80% of the carbohydrates in Myco 1 and Myco 2 are dietary fibre. This finding aligns with the production of mycoprotein biomass, which leads to a reduced carbohydrate content, as fungi utilize carbohydrates during their growth, irrespective of the fungal species used [52,53,54].

Regarding the fat content, significant differences were observed among the samples analysed: Myco 1 (3.2 ± 0.029%), Myco 2 (2.2 ± 0.016%), and *Durvillaea* spp. (0.43 ± 0.022%) [46]. Compared with that in the seaweed biomass, the crude fat content in Myco 1 increased significantly by 644.1% (Table 1). Notably, the fat in Myco 1 (Table 2) is predominantly composed of mono- and polyunsaturated fatty acids, including eicosapentaenoic acid (omega-3), linoleic acid (omega-3), and other omega-6 fatty acids.

The nutritional value of this mycoprotein (Myco 1 and 2) is highly satisfactory. These results are comparable to the nutritional characteristics (fat, carbohydrate, and fibre contents) found in commercial mycoproteins produced by Quorn^®^, Mycotechnology^®^, Micorena^®^, and Fermotein^®^, among others [13,55]. Additionally, previous studies have shown that Myco 1 and 2 are free from aflatoxin and ochratoxin mycotoxins and that they do not accumulate heavy metals (Hg, As, Pb, and Cd). These findings indicate that these mycoproteins are excellent sources of protein with a low energy content and considerable fibre content.

Furthermore, the amino acid content and profile of these mycoproteins (Myco 1 and 2) were superior to those of *Durvillaea* spp. As shown in Table 3, the total amino acid content of Myco 1 and Myco 2 was significantly greater than that of *Durvillaea* spp. (*p* < 0.05). Myco 1 had the highest total concentration of essential amino acids (165 mg/g). All essential amino acids in Myco 1 significantly increased (*p* < 0.05), with up to a 2.8-fold increase compared to that in *Durvillaea* spp. (Table 3). Significant differences (*p* < 0.05) in the methionine, serine, and glycine contents were also detected between the Myco 1 and Myco 2 groups (Table 3). Additionally, the content of nonessential amino acids increased in both Myco 1 and Myco 2 as compared to that in *Durvillaea* spp., with serine, glycine, aspartic acid, and glutamic acid showing notable increases. Specifically, aspartic acid and glutamic acid in Myco 2 increased up to 8.8-fold and 2.1-fold, respectively (Table 3).

Similar increases in amino acid levels through fermentation with different microorganisms have been reported in other studies [56]. According to the literature, these increases in amino acids correspond to the action of transaminases produced by fungi during fermentation, which facilitate amino acid synthesis or transamination [57]. The nutrient preferences of fungi, including their amino acid content, have been studied extensively in the brewing industry, revealing a clear order of preference. The metabolic incorporation of amino acids from the extracellular solution depends on the internal nitrogen supply and demand, as well as their availability in the external environment. Consequently, the intracellular pool in fungi typically consists of core amino acids such as glutamate, aspartic acid, and alanine, with smaller amounts of arginine, lysine, and histidine [58]. Moreover, aspartic acid and glutamic acid are crucial because they contribute to the umami flavour, creating a new natural flavouring, which has been documented in other fungal studies [59]. These mycoproteins contain all essential amino acids, and the literature reports a digestibility-corrected amino acid score of 0.996 for other mycoproteins, indicating that they are a complete protein source with a bioavailability similar to that of dairy milk and superior to that of wheat-based or soy-based proteins [60].

### 3.2. Estimation of Beta-Glucan and Ergosterol Contents

**Figure 1:**
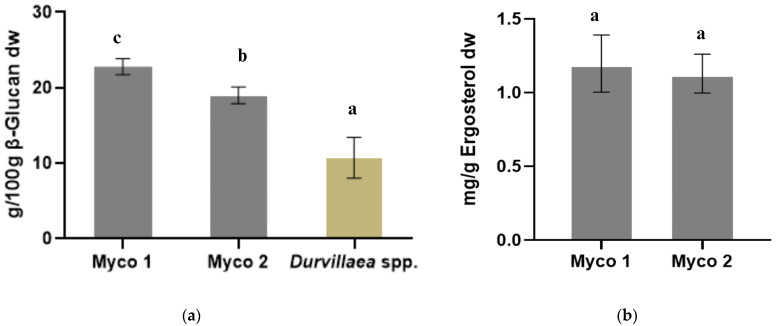
Beta-glucan content (**a**) and ergosterol content (**b**) in Myco 1, Myco 2, and *Durvillaea* spp. samples. Values are presented as mean ± SD. Bars with different letters indicate significant differences (*p* < 0.05). All values are based on dry weight (dw) analysis. The different bar colours are used solely for visual distinction between the data for Myco and *Durvillaea* spp. and do not represent any additional variable.

The β-glucan content in Mycoseaweed’s products and *Durvillaea* spp. showed significant variations. The values ranged from 19.01 ± 0.91 g/100 g (dw) in Myco 1 to 22.79 ± 0.87 g/100 g (dw) in Myco 2 (Figure 1a). These levels were significantly greater (*p* < 0.05) than those of *Durvillaea* spp., which had a β-glucan content of 10.73 ± 2.2 g/100 g dry weight (Figure 1a).

Beta-glucans are naturally occurring polysaccharides found across various organisms, including bacteria, algae, plants such as cereal seeds, and both micro- and macrofungi [61]. Their content varies notably among fungal cultivars within the Ascomycete and Basidiomycete classes and is influenced by genus, species, and whether the analysis involves the mycelium or fruiting body. Ascomycete mycelia can contain beta-glucans ranging from 5% to 55%, for example, in *Aspergillus niger*, it accounts for up to 59%, while in *Cordyceps sinensis*, it accounts for up to 10.9% [36]. In Basidiomycetes, both wild and commercially grown species such as *Lentinula*, *Ganoderma*, and *Pleurotus*, the beta-glucan content can vary from 2% to 50%, depending on whether the mycelium or different parts of the fruiting bodies (pileus and stipe) are analysed [62]. Furthermore, in fungi, substrate variations during cultivation also significantly impact the total glucan content, including both alpha- and beta-glucans [6]. On the other hand, studies on seaweed show beta-glucan levels ranging from 0.5% to 15%, depending on the part of the algae analysed and the collection season. Specifically, *Durvillaea antarctica* has been shown to contain between 3.7% and 14.5% beta-glucan [63]. Beta-glucans, key components of fungal cell walls, have been extensively studied in recent years, and their immunomodulatory, anti-inflammatory, and cardiovascular protective effects have been explored in clinical trials, often using fungus-derived beta-glucans or concentrated extracts for potential drug discovery [64,65].

The ergosterol levels in the dry biomass were quantified, and the levels of Mycoseaweed mycoprotein ranged from 1.13 ± 0.11 mg/g to 1.20 ± 0.16 mg/g for Myco 1 and 2, respectively (Figure 1b). Ergosterol is commonly used as an indirect method to estimate fungal biomass in various studies. Ergosterol, also known as calciferol (C_28_H_44_O), is a key component of the fungal cell membrane, representing 0.7 to 1% of fungal dry matter [66]. The ergosterol findings in our samples are consistent with those reported in other studies, where the ergosterol content in various fungi ranged from 0.4 to 4.5 mg/g in mycelia (mycoprotein) or fruiting bodies [67]. Factors such as the medium and extraction methods can influence ergosterol levels, with lower values potentially due to extraction inefficiencies [68]. Ergosterol, a precursor to vitamin D, is linked to beneficial effects on the cardiovascular system and lipid metabolism [69]. There was no ergosterol detected in *Durvillaea* spp. because ergosterol is exclusively found in fungi, with only trace amounts in certain bacteria, protozoa, and cyanobacteria, and is absent in plants, animals, and other organisms such as algae [70].

### 3.3. Phenolic Compounds and Bioactivity In Vitro

Given their potent antioxidant properties, the total phenolic content (TPC) and specific phenolic compounds were analysed in Mycoseaweed’s mycoprotein and *Durvillaea* spp. The TPC of the mycoprotein significantly exceeded that of *Durvillaea* spp. (*p* < 0.05) (Table 4), with Myco 1 and 2 showing a 26.5% and 59% increase in the phenolic content compared to the seaweed sample, respectively.

These findings align with previous research indicating that the phenolic content can be enhanced through fermentation or bioconversion processes using fungal enzymes on brown algae or agricultural waste [20,52,71]. The increase in phenolic content in fermented seaweed biomass is linked to the presence of storage polysaccharides such as alginate and fucoidan in brown seaweed cell walls, which are associated with proteins and phenolic compounds. Fungal enzymes can break down these cell walls, releasing sugars, proteins, and phenolic compounds and enhancing bioavailability [72,73]. Given their health benefits, including antioxidant properties, phenolic compounds from fungi are increasingly recognized as functional food ingredients [74]. Qualitative and quantitative analyses of eight phenolic acids and derivatives (4-OH-benzoic acid, vanillic acid, gallic acid, 3-hydroxytyrosol, catechin, epicatechin, salicylic acid, and pinocembrin) were conducted on the studied mycoproteins (Table 5). Both the Myco 1 and Myco 2 samples contained 4-hydroxybenzoic acid, vanillic acid, epicatechin, and pinocembrin. Myco 2 exhibited the highest total phenolic content (TPC) of 1.32 ± 0.03 mg/g sample dry weight, which was influenced by 3-hydroxytyrosol (7.16 ± 0.688 µg/g), 4-OH-benzoic acid (3.1 ± 0.01 µg/g), and 3,4-dihydroxyphenylglycol (16.48 ± 0.851 µg/g). Myco 1 also had a substantial phenolic acid content (1.05 ± 0.09 mg/g sample dry weight), predominantly 3,4-dihydroxyphenylglycol. 

Comparative studies indicate that mycoproteins from various microorganisms contain a diverse array of bioactive metabolites, including flavonoids and phenolic compounds [75]. The literature reported various phenolic acids in different kinds of mushrooms, with concentrations ranging from 2.79 to 53.13 mg GAE/g extract [76]. These included 4-OH-benzoic, vanillic, and salicylic acids, with the latter present in smaller quantities. Moreover, seaweeds are known for their richness in diverse polyphenolic compounds, such as gallic acid, p-hydroxybenzoic, vanillic, syringic acids, and protocatechins. These often form complexes with sugars to produce tannins, complicating their detection [77]. This study revealed a greater content of pinocembrin (4.091 ± 2.612 µg/g) in *Durvillaea* spp. Interestingly, Myco 1 and Myco 2 exhibited lower concentrations of pinocembrin (0.485 and 0.469 µg/g) than those in this marine alga. Consequently, some of the phenolic acids found in this mycoprotein could be derived from *Durvillaea* spp.

Biological activities, such as antioxidant and antihyperglycemic activities, can be related to the phenolic acid content in fungi. As shown in Table 4, the Trolox equivalent antioxidant capacity (TEAC) of Myco 1, Myco 2, and *Durvillaea* spp. did not vary significantly. The results indicated that Myco 2 exhibited the highest antioxidant activity in vitro (25.5 ± 1.5 µmol TE/g dw). This activity was 18.6% higher than that of Myco 1 (21.5 ± 1.7 µmol TE/g dw) and 12.3% higher than that of *Durvillaea* spp. (22.7 ± 1.5 µmol TE/g dw).

In this study, the antioxidant activity in vitro was measured with an improved antioxidant assay kit (Sigma Aldrich), in which Cu^2+^ is reduced by an antioxidant to Cu^+^. The resulting Cu^+^ forms a specified coloured complex with a dye reagent. Metal chelation is a crucial antioxidant mechanism that prevents metallic ions (Cu^2+^ and Cu^+^) from converting lipid hydroperoxides into free radicals. Polyphenols, multifunctional antioxidant metabolites, exhibit strong metal chelating properties, highlighting their importance as antioxidant compounds in food systems [78,79]. Furthermore, studies have demonstrated that extraction methods significantly influence the quality and quantity of polyphenols, thereby altering their antioxidant activity. In this context, polyphenol extracts from two wild edible *Melanoleuca* mushrooms were investigated by Bahadori et al. [79], with reducing powers ranging from 1.7 to 41 μmol TE/g dw. Similarly, edible mushrooms from the genus *Ganoderma* exhibited values between 3.5 and 84.8 μmol TE/g dw [80]. Our results indicate that studied mycoproteins exhibit good antioxidant activity (Table 4) compared to some commercial mushroom extracts. For instance, *Ganoderma*, *Lentinula*, *Hericium*, and *Trametes* showed TEAC values of 23, 30, 26, and 36 μmol TE/g, respectively [81]. Regarding the TEAC of *Durvillaea* spp., our results did not show a considerable difference compared to those of the mycoproteins (Table 4). Previous studies on *Durvillaea antarctica* have reported higher TEAC values (300–500 μmol TE/g) than those presented in our work [82]. These discrepancies between the observed results in different antioxidant assays can be attributed to variations in the extraction conditions and measurement methodologies. Therefore, to optimize the conditions for TEAC, future research should explore additional extraction and quantification methods.

While multiple digestive enzymes play a role in starch breakdown, alpha-glucosidase is frequently utilized in established enzymatic models to evaluate potential agents for hyperglycaemia management [83]. For alpha-glucosidase inhibition activity, the IC_50_ values calculated for each sample are shown in Table 4. Myco 1 exhibited the highest α-glucosidase inhibitory activity (IC_50_ = 1.65 mg/mL), followed by Myco 2 (IC_50_ = 2.21 mg/mL) and *Durvillaea* spp. (IC_50_ = 5.263 mg/mL). The inhibitory activity against α-glucosidase can be attributed to the phenolic compound content in the mycoprotein and seaweed biomass [19]. Notably, in this study, Myco 2 contained the greatest amount of total phenolics (Table 4). Thus, other bioactive constituents, such as the unsaturated fatty acids present in Myco 1 (Table 2), may have contributed to the potent inhibitory effect on alpha-glucosidase activity [84]. The IC_50_ values for anti-alpha-glucosidase activity from mycoprotein in this study were greater than those of some commercial and wild mushroom extracts reported by Wunjuntuk et al. [85], where they inhibited alpha-glucosidase (12.9–203 mg/mL) from ethanol and hexane extracts of various mushrooms, including *Lentinula edodes*, *Schizophyllum commune*, *Pleurotus djamor*, and *Lentinula flavidulus.*

Pancreatic lipase hydrolyses triglycerides into one glycerol molecule and three fatty acids and breaks down ingested triacylglycerols into 2-monoacylglycerol and fatty acids in the small intestine [86]. For lipase inhibition, Table 4 shows the IC_50_ results for Myco 1 and Myco 2, indicating significantly lower inhibitory activity than that of *Durvillaea* spp. (IC_50_ = 0.32 mg/mL). These results are better than those reported by Jeong et al. [87], where the IC_50_ values of ethanol extracts from the seaweeds *Myagropsis myagroides* and *Sargassum muticum* showed activity at 5 mg/mL. In another study, extracts from the seaweeds *Kappaphycus alvarezii*, *Kappaphycus striatus*, and *Eucheuma denticulatum* produced a decrease in lipase activity to 3.8 mg/mL [88]. Moreover, the lipase inhibition results obtained in this study were similar to those obtained for the popular cultivated mushroom *Agaricus bisporus* (1.0 mg/mL) [89]. Therefore, Myco 1 and Myco 2 flour exhibited notable glucosidase and lipase inhibitory activities, indicating that whole seaweed powder could serve as a dietary supplement for managing hyperglycaemia and weight without requiring ethanol extraction.

### 3.4. Metabolomic Differences between Myco 1, Myco 2, and Durvillaea spp.

The chemical profiles of mycoprotein (Myco 1 and 2) and *Durvillaea* spp. extracts were organized in a classical molecular networking (CMN), and dereplication was conducted based on ESI-(+)-HRMS/MS analysis and GNPS and relevant databases. A total of 231 metabolites were annotated, with 69 upregulated, 102 downregulated, and 60 insignificant. These results from nontargeted metabolomics revealed markedly different metabolomes between the mycoprotein (Myco 1 and Myco 2) and *Durvillaea* spp. These metabolites exhibited distinct clustering patterns in the principal component analysis (PCA), with the mycoprotein and *Durvillaea* spp. clearly discriminated along PC1 (52.27%). Additionally, the metabolic profiles for Myco 1 and 2 were distinctly separated along PC2 (42.73%) (Figure S2; Supplementary Materials).

Dereplication tools can serve as valuable complementary devices for detecting edible compounds and quantifying dietary nutrients such as fatty acids and steroids. Therefore, we performed dereplication of the MeOH extracts using the GNPS molecular network, employing molecular fragmentation data obtained via *tandem* mass spectrometry [90]. The platform analyses each *tandem* MS spectrum and compares the molecular fragments and exact masses to a spectral database. Based on user-defined parameters, the GNPS platform can annotate molecules and create a molecular networking, grouping them according to similarities [44]. GNPS annotated various compounds, including monoacylglycerols, organic acids, and fatty acids (Table 6).

The spectral families generated by the platform consisted mostly of fatty acids (Figure S3; Supplementary Materials). Notably, monoacylglycerols containing unsaturated fatty acids were also annotated. Monoacylglycerols such as monoelaidin and monolinolenin have functional properties as emulsifiers and texturizers and are rich in omega-3 fatty acids, making them beneficial for cardiovascular health, reducing inflammation, and exhibiting bactericidal activities [91]. Additionally, 9-octadecenamide, known as oleamide, is a fatty acid amide with anti-inflammatory and neuroprotective properties [92]. Unsaturated fatty acids are crucial for human health because they regulate cell physiology and reduce cholesterol levels [93]. Since higher animals cannot synthesize all necessary fatty acids, they must obtain these essential nutrients through their diet [94]. Given the abundance of unexplored metabolites in mycoproteins, particularly those produced through the bioconversion of seaweeds, their biological, nutraceutical, and medicinal properties, as well as the discovery of bioactive molecules, remain understudied. Therefore, future research should include bioactivity tests using crude extracts using different solvents and fractions to identify and characterize metabolites with biological activity from novel mycoproteins or alternative proteins.

### 3.5. Emulsion Capacity (EC) and Emulsion Stability (ES)

**Figure 2:**
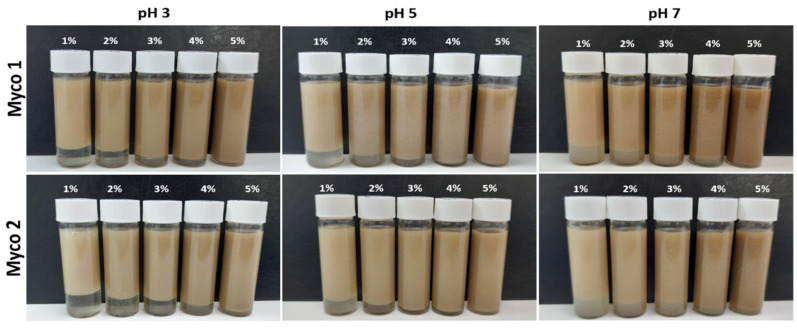
Images of the O/W emulsions stabilized by Myco 1 and Myco 2 at different concentrations (1–5%, *w*/*w*).

Emulsification is one of the most important processes in the manufacturing of formulated foods. To explore the potential applications of mycoproteins produced by Mycoseaweed as emulsifiers, the effect of sample type (Myco 1 and Myco 2) and concentration on the emulsion capacity (EC) and emulsion stability (ES) were assessed (Table 7). The emulsions were prepared and stabilized with different concentrations of each mycoprotein sample (1–5% *w*/*v*), and sunflower oil was used as the dispersion phase (Figure 2).

For both Myco 1 and Myco 2, the results indicated that the emulsifying capacity depended on the concentration. The results suggest that the emulsion stability improves with a gradual increase in the concentration of the mycoprotein. Furthermore, as visually depicted in Figure 2, there were no apparent signs of macroscopic oil leakage at the top of the tubes, confirming the good stability of the emulsion. Additionally, a slight improvement in the EC values was observed at pH 3, where values of approximately 100% were achieved between 4% and 5%. In addition, a slight improvement in the EC values was observed at pH 3, where the values were slightly greater than those of the emulsions stabilized at pH 3 and 7. This improvement could be attributed to the fact that the ionic compounds present in the mycoprotein may be influenced by pH changes, which enhances their interfacial activity at this pH. According to Pacwa-Plociniczak et al. [95], bioemulsifiers containing amphiphilic polysaccharides, proteins, lipopolysaccharides, lipoproteins, or complex mixtures of these biopolymers are effective at stabilizing O/W emulsions. The presence of proteins and polysaccharides in the mycoprotein may play a relevant role in the formation and stabilization of emulsions due to its amphiphilic nature. Moreover, one criterion used to identify a bioemulsifier is its ability to maintain at least 50% of the original emulsion volume after 24 h [96]. Consequently, both Mycoseaweed’s mycoprotein samples can be included in this family of compounds.

**Figure 3:**
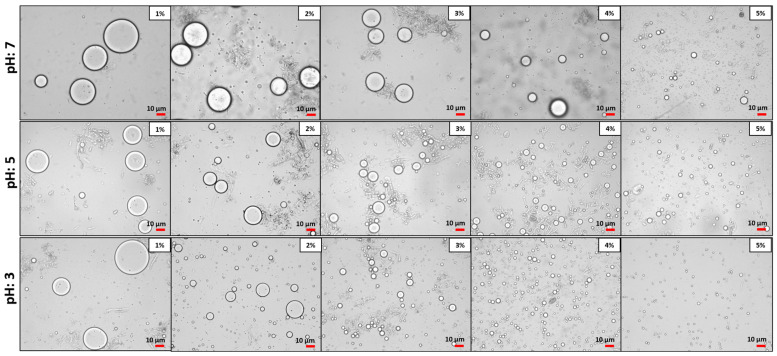
Optical micrographs of the O/W Pickering emulsions stabilized at different Myco 1 concentrations (1.0–5.0%, *w*/*w*). The images were acquired at 40× magnification.

**Figure 4:**
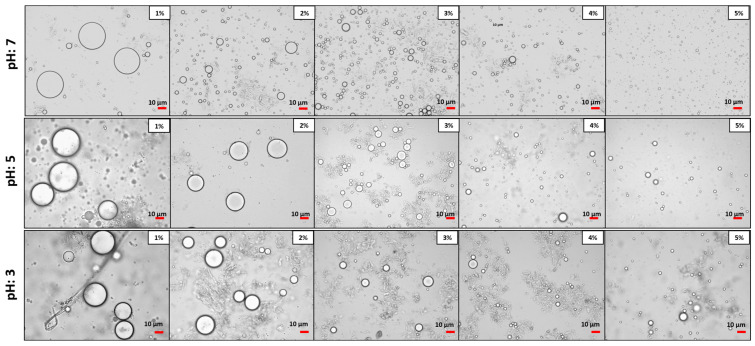
Optical micrographs of the O/W Pickering emulsions stabilized at different Myco 2 concentrations (1.0–5.0%, *w*/*w*). The images were acquired at 40× magnification.

Figure 3 and Figure 4 show micrographs of the emulsions stabilized by Myco 1 and Myco 2. The images show that the droplet size of the O/W emulsions decreased as the sample concentration increased from 1 to 5% (*w*/*w*) at a fixed oil phase concentration (50%, *w*/*w*). Additionally, the images indicate no significant differences in the droplet sizes with varying mycoprotein concentrations among the different type of samples (Myco 1 and Myco 2).

According to Burgos-Díaz et al. [97], low emulsifier concentrations result in larger oil droplets. This occurs because there is insufficient emulsifier to fully cover the interface of the newly formed oil droplets during homogenization, leading to coalescence and even oil separation in the emulsion. Conversely, at higher concentrations, sufficient surface coverage can be achieved, resulting in smaller droplets. Burgos-Díaz et al. [97] observed a similar qualitative trend in O/W emulsions stabilized by agri-food byproducts as emulsifiers. When the particle emulsifier concentration rises at a constant droplet fraction, a larger interfacial area is covered, resulting in smaller droplets.

## 4. Conclusions

In conclusion, the bioconversion of *Durvillaea* spp. results in a mycoprotein with high-quality protein, significant nutritional and functional value, and prebiotic and nutraceutical potential due to the production of unique bioactive compounds. This study demonstrates, for the first time, that this novel mycoprotein not only contains all essential amino acids and has a low fatty acid content but also exhibits high antioxidant activity and bioemulsifying properties, positioning it as a promising alternative for the development of functional and nutraceutical foods. Additionally, the observed increases in protein, amino acids, and β-D-glucans highlight the superior nutritional profile of this mycoprotein compared to its source material. Its anti-alpha-glucosidase and anti-lipase activities further underscore its potential health benefits, making it an excellent candidate for use in health-promoting dietary applications. Looking ahead, future research could explore the scalability of this bioconversion process for industrial production and investigate the long-term health effects of incorporating this mycoprotein into various dietary regimes. Additionally, further studies could examine the potential for this mycoprotein to serve as a sustainable alternative protein source in diverse culinary applications.

## Figures and Tables

**Table 1:** Nutritional composition of Myco 1, Myco 2 (g per 100 g of dw), and *Durvillaea* spp. (g per 100 g dw).

Sample	Nutritional Composition (% Dry Weight)				
	Ash	Moisture	Protein	Carbohydrate	Fat
Myco 1	12.3 ± 0.12 ^a^	10.2 ± 0.083 ^a^	36.5 ± 0.189 ^c^	38.2 ± 0.260 ^a^	3.2 ± 0.029 ^c^
Myco 2	13.5 ± 0.133 ^a^	11.2 ± 0.061 ^a^	33.8 ± 0.170 ^b^	39.1 ± 0.113 ^a^	2.2 ± 0.016 ^b^

The data are expressed as the means ± standard deviations (SDs). ^a–c^ Different lowercase letters indicate significant differences (*p* < 0.05) in the parameter evaluated.

**Table 2:** Principal Fatty Acid Profile of Fat in Myco 1 (mg/100 g dry weight).

Principal Fatty Acids in Myco 1	mg/100 g (dw)
**Saturated fatty acids**	491.9
**C16:0 (Palmitic acid)**	326.1
**C18:0 (Stearic acid)**	113.4
**Monounsaturated fatty acids**	374.8
**9c-18:1**	314.8
**Polyunsaturated fatty acids**	815.9
**C18:2n-6 (Linoleic acid)**	703.1
**C18:3n6 (gamma-Linoleic acid)**	8.5
**C18:3n3 (alpha-Linoleic acid)**	34.8
**C18:4n3 (derived from omega-3 fatty acids)**	15.9
**C20:4n3 (derived from omega-3 fatty acids)**	1.5
**C20:5n3 EPA (Eicosapentaenoic acid omega-3 fatty acids)**	13.3
**C20:4n6 (derived from omega-6 fatty acids)**	1.0

**Table 3:** Amino acid profiles of Myco 1, Myco 2, and *Durvillaea* spp.

	Amino Acid (mg/g of Sample dw)	Myco 1	Myco 2	*Durvillaea* spp.
Essential amino acids	Histidine	10.98 ± 0.31 ^b^	9.57 ± 0.31 ^b^	1.16 ± 0.02 ^a^
	Threonine + Arginine	36.91 ± 1.12 ^b^	33.27 ± 1.55 ^b^	11.9 ± 0.26 ^a^
	Tyrosine	11.37 ± 0.14 ^b^	11.97 ± 0.11 ^b^	9.32 ± 0.18 ^a^
	Alanine	21.26 ± 0.51 ^b^	15.20 ± 0.25 ^b^	2.90 ± 0.06 ^a^
	Methionine	13.02 ± 0.26 ^c^	14.80 ± 0.10 ^b^	6.09 ± 0.15 ^a^
	Valine	16.83 ± 0.30 ^b^	14.88 ± 0.18 ^ab^	11.47 ± 0.14 ^a^
	Phenylalanine	14.66 ± 2.06 ^b^	14.06 ± 0.74 ^b^	3.41 ± 0.08 ^a^
	Leucine	20.92 ± 0.15 ^b^	20.20 ± 0.58 ^b^	6.07 ± 0.12 ^a^
	Isoleucine	19.91 ± 0.36 ^b^	19.00 ± 1.4 ^b^	5.62 ± 0.13 ^a^
	Essential sum	165.09	152.99	58.04
Nonessential	Serine	17.62 ± 0.04 ^c^	13.67 ± 0.22 ^b^	3.15 ± 0.02 ^a^
	Aspartic acid	34.65 ± 0.83 ^b^	25.26 ± 0.41 ^b^	3.93 ± 0.07 ^a^
	Glutamic acid	15.54 ± 0.45 ^b^	13.65 ± 0.45 ^b^	7.24 ± 0.15 ^a^
	Glycine	12.45 ± 0.30 ^c^	9.30 ± 0.30 ^b^	4.66 ± 0.13 ^a^
	Total sum	246.10 ^c^	214.90 ^b^	77.04 ^a^

The data are expressed as the means ± standard deviations (SDs). ^a–c^ Different lowercase letters indicate significant differences (*p* < 0.05) in the parameter evaluated.

**Table 4:** Total phenolic compounds, antioxidant activity, and enzymatic inhibition capacity of Myco 1, Myco 2, and *Durvillaea* spp.

Sample	Total Phenolic (mg GAE/g)	Antioxidant Activity (µmol TE/g)	α-Glucosidase Inhibition IC 50 (mg/mL)	Lipase Inhibition IC 50 (mg/mL)
Myco 1	1.05 ± 0.09 ^b^	21.5 ± 1.7 ^a^	1.65 ± 75.2 ^a^	1.05 ± 34.2 ^b^
Myco 2	1.32 ± 0.03 ^c^	25.5 ± 1.5 ^a^	2.21 ± 42.1 ^b^	1.19 ± 77.6 ^c^
*Durvillaea* spp.	0.83 ± 0.06 ^a^	22.7 ± 1.5 ^a^	5.26 ± 85.3 ^c^	0.32 ± 81.5 ^a^

GAE: Galic acid equivalent; TE: Trolox equivalent. ^a–c^ Different lowercase letters indicate significant differences (*p* < 0.05) in the corresponding parameter.

**Table 5:** Phenolic content (μg/g sample dw) of Myco 1, Myco 2, and *Durvillaea* spp. Analysis performed on a dry weight (dw) basis.

	Concentration ± SD (µg/g Sample dw)		
Phenolic Acids and Derivatives	Myco 1	Myco 2	*Durvillaea* spp.
**4-Hydroxybenzoic acid**	2.921 ± 0.050	3.170 ± 0.019	N.D
**Vanillic acid**	0.250 ± 0.014	0.240 ± 0.022	N.D
**3,4-dihydroxyphenylglycol**	38.826 ± 0.703	16.485 ± 0.851	13.747 ± 1.21
**Gallic acid**	N.D	0.685 ± 0.34	0.410 ± 0.005
**3-Hydroxytyrosol**		7.16 ± 0.688	N.D
**Flavan-3-ol**			
**Catechin**	0.056 ± 0.001	N.D	N.D
**Epicatechin**	0.552 ± 0.022	0.437 ± 0.012	N.D
**Flavones and Flavonols**			
**Salicylic acid**	N.D	0.483 ± 0.015	N.D
**Pinocembrin**	0.485 ± 0.008	0.469 ± 0.058	4.091 ± 2.612

N.D: Not detected.

**Table 6:** Dereplication of compounds from Myco 1, Myco 2, and *Durvillaea* spp. based on GNPS spectral library matching.

Sample	Compound	Classification	Molecular Formula
Myco 1	Monoelaidin	Monoacylglycerol	C_21_H_40_O_4_
	Monolinolenin	Monoacylglycerol	C_21_H_34_O_4_
	Linoleoylglycerol	Monoacylglycerol	C_21_H_38_O_4_
	Oxobutanoic acid	Organic acids	C_4_H_6_O_3_
Myco 2	Phytomonic acid	Fatty acids	C_16_H_30_O_2_
	9-octadecenamide (Oleamide)	Fatty acids	C_18_H_35_NO
	N-Cyclohexanecarbonylpentadecylamine		C_22_H_43_NO
	13-docosenamide (Erucamide)	Fatty acids	C_22_H_43_NO
*Durvillaea* spp.	Palmitoyl	Fatty acids	C_16_H_31_O_2_

**Table 7:** Emulsifying properties of Myco 1 and Myco 2 at different pH levels.

Concentration Myco 1	pH 7	pH 5	pH 3
1%	EC_0_: 79.93 ± 0.40 ^a,A^ ES: 88.79 ± 1.06 ^a,A^	EC_0_: 82.36 ± 0.56 ^b,A^ ES: 95.93 ± 0.90 ^b,A^	EC_0_: 79.87 ± 0.81 ^a,A^ ES: 95.18 ± 1.05 ^b,A^
2%	EC_0_: 82.38 ± 2.51 ^ab,A^ ES: 92.57 ± 2.50 ^a,AB^	EC_0_: 86.80 ± 1.59 ^b,B^ ES: 98.47 ± 1.50 ^b,AB^	EC_0_: 80.64 ± 2.51 ^a,A^ ES: 97.59 ± 2.50 ^b,AB^
3%	EC_0_: 87.21 ± 2.55 ^a,B^ ES: 93.86 ± 3.43 ^a,AB^	EC_0_: 93.16 ± 2.75 ^b,C^ ES: 97.07 ± 2.00 ^a,AB^	EC_0_: 89.47 ± 2.25 ^ab,B^ ES: 97.06 ± 1.68 ^a,AB^
4%	EC_0_: 92.84 ± 2.57 ^a,C^ ES: 94.21 ± 1.94 ^a,B^	EC_0_: 96.59 ± 1.51 ^a,D^ ES: 98.96 ± 0.07 ^b,B^	EC_0_: 92.94 ± 2.61 ^a,B^ ES: 96.03 ± 1.00 ^a,AB^
5%	EC_0_: 100 ± 0,00 ^a,D^ ES: 92.09 ± 2.60 ^a,B^	EC_0_: 100 ± 0.00 ^a,E^ ES: 95.08 ± 3.00 ^ab,B^	EC_0_: 98.01 ± 1.74 ^a,C^ ES: 98.63 ± 1.52 ^b,B^
**Concentration** **Myco 2**	**pH 7**	**pH 5**	**pH 3**
**1%**	EC_0_: 79.65 ± 3.06 ^ab,A^ ES: 87.97 ± 2.63 ^a,A^	EC_0_: 84.01 ± 4.00 ^b,A^ ES: 98.72 ± 0.63 ^b,C^	EC_0_: 75.55 ± 1.27 ^a,A^ ES: 97.49 ± 2.50 ^b,A^
**2%**	EC_0_: 85.97 ± 2.00 ^b,B^ ES: 86.80 ± 2.78 ^a,A^	EC_0_: 89.79 ± 2.03 ^b,A^ ES: 98.85 ± 0.79 ^b,C^	EC_0_: 79.61 ± 3.08 ^a,AB^ ES: 98.44 ± 2.14 ^bA^
**3%**	EC_0_: 87.29 ± 2.53 ^a,B^ ES: 87.92 ± 2.74 ^a,A^	EC_0_: 94.80 ± 2.55 ^b,B^ ES: 98.21 ± 1.06 ^b,BC^	EC_0_: 83.05 ± 2.68 ^a,B^ ES: 98.84 ± 1.61 ^b,A^
**4%**	EC_0_: 93.47 ± 3.01 ^ab,C^ ES: 90.68 ± 2.52 ^a,A^	EC_0_: 97.58 ± 1.51 ^b,B^ ES: 96.05 ± 1.00 ^b,AB^	EC_0_: 88.63 ± 3.18 ^a,C^ ES: 99.63 ± 0.64 ^c,A^
**5%**	EC_0_: 93.86 ± 3.43 ^a,C^ ES: 96.07 ± 2.00 ^a,B^	EC_0_: 100 ± 0.00 ^b,B^ ES: 95.65 ± 2.51 ^a,A^	EC_0_: 95.75 ± 2.54 ^ab,D^ ES: 99.31 ± 1.20 ^a,A^

Values are presented as mean ± SD. EC: emulsifying capacity (%); ES: emulsifying stability (%). Different lowercase letters in the same row denote significant differences in each concentration (*p* < 0.05). Different capital letters in the same column denote significant differences in each pH (*p* < 0.05). All values are based on dry weight (dw) analysis.

## Data Availability

The original contributions presented in the study are included in the article/Supplementary Material, further inquiries can be directed to the corresponding author.

## Supplementary Materials

The following supporting information can be downloaded at: https://www.mdpi.com/article/10.3390/foods13152376/s1, Figure S1: Detailed schematic diagram illustrating the workflow and key outcomes of the study; Figure S2: Principal component analysis (PCA) score plots of the differential metabolites from Myco 1, Myco 2, and *Durvillaea* spp., analysed using UHPLC-QqTOF-MS/MS; Figure S3: Spectral families generated by the platform GNPS molecular network.
