# Supplementary material for: Comprehensive Nutritional and Functional Characterization of Novel Mycoprotein Derived from the Bioconversion of Durvillaea spp
Source: Foods. 2024 Jul 27;13(15):2376. doi: 10.3390/foods13152376 (PMC11312218; doi:10.3390/foods13152376)

SUPPLEMENTARY MATERIALS

Comprehensive Nutritional and Functional Characterization of Novel Mycoprotein Derived from the Bioconversion of *Durvillaea* spp.

Catalina Landeta-Salgado [1,*], Nicolás Salas-Wallach [1], Javiera Munizaga [1], María Paz González-Troncoso [1],
César Burgos-Díaz [2], Lhaís Araújo-Caldas [3], Patricia Sartorelli [3], Irene Martínez [1] and María Elena Lienqueo [1]

[1] Department of Chemical Engineering, Biotechnology, and Materials, Center for Biotechnology and Bioengineering (CeBiB), University of Chile, Beauchef 851, Santiago 8370456, Chile; n.salas.wallach@gmail.com (N.S.-W.); jmmunizaga@uc.cl (J.M.); imartinez@ing.uchile.cl (I.M.); mlienque@ing.uchile.cl (M.E.L.)
[2] Agriaquaculture Nutritional Genomic Center, CGNA, Temuco 4780000, Chile; cesar.burgos@cgna.cl
[3] Instituto de Ciências Ambientais, Químicas e Farmacêuticas, Universidade Federal de São Paulo, Diadema Campus, Sao Paulo 09913-030, SP, Brazil; lhaisaraujocaldas@gmail.com (L.A.-C.); psartorelli@unifesp.br (P.S.)
* Correspondence: cmlandeta@uc.cl; Tel.: +56-978864013

Figure S1 – Detailed schematic diagram illustrating the workflow and key outcomes of the study.

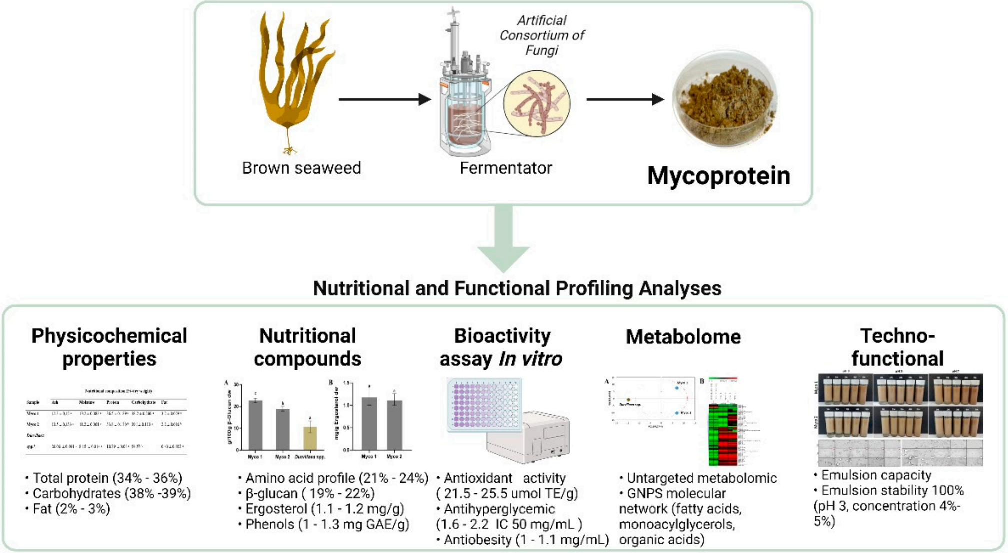

Figure S2 – Principal component analysis (PCA) score plots of the differential metabolites from Myco 1, Myco 2, and *Durvillaea* spp., analysed using UHPLC-QqTOF-MS/MS.

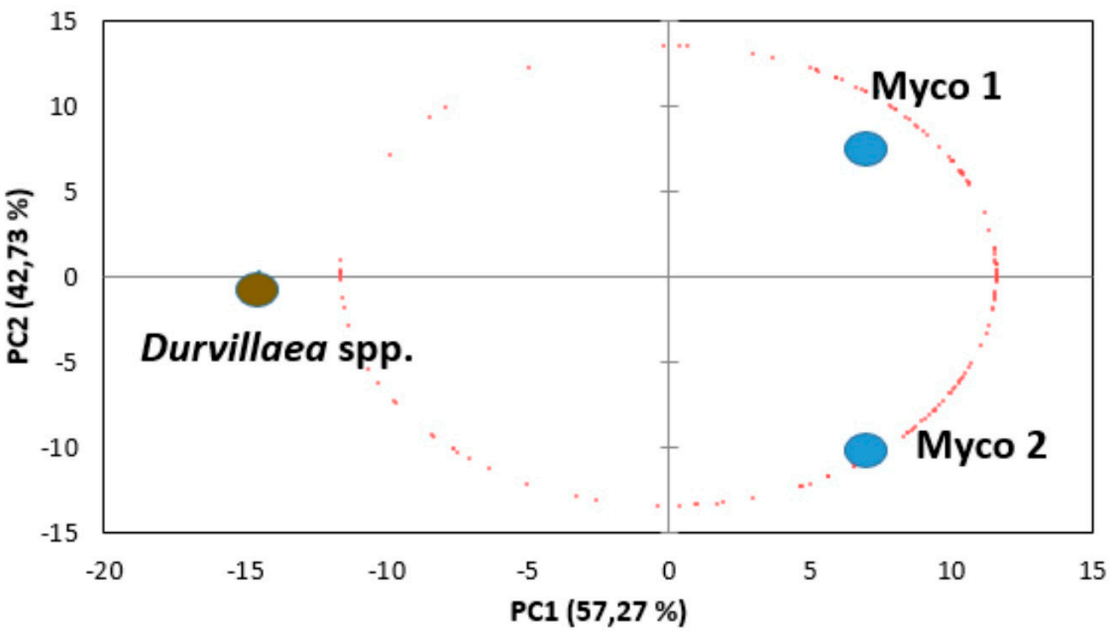

Figure S3 –Spectral families generated by the platform GNPS molecular network

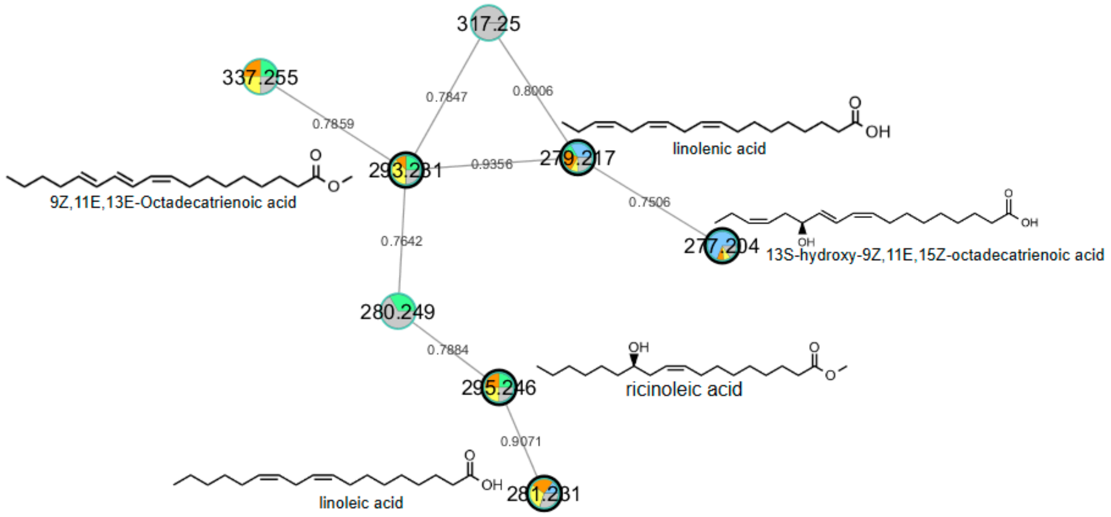

Supplement: Supplementary file 1 [file foods-13-02376-s001.zip › foods-3108891-supplementary.pdf]
